# Soccer-Related Concussions Among Swedish Elite Soccer Players: A Descriptive Study of 1,030 Players

**DOI:** 10.3389/fneur.2020.510800

**Published:** 2020-09-23

**Authors:** Sofie Hänni, Fredrik Vedung, Yelverton Tegner, Niklas Marklund, Jakob Johansson

**Affiliations:** ^1^Department of Neuroscience, Neurosurgery, Uppsala University, Uppsala, Sweden; ^2^Department of Surgical Sciences, Anesthesiology, Uppsala University, Uppsala, Sweden; ^3^Division of Health, Medicine and Rehabilitation, Department of Health Sciences, Luleå University of Technology, Luleå, Sweden; ^4^Department of Clinical Sciences Lund, Neurosurgery, Skane University Hospital, Lund University, Lund, Sweden

**Keywords:** concussion, soccer-related concussion, soccer, SCAT3, sex differences

## Abstract

**Objective:** There are growing concerns about the short- and long-term consequences of sports-related concussion, which account for about 5–9% of all sports injuries. We hypothesized there may be sex differences in concussion history and concussion-related symptoms, evaluated among elite soccer players in Sweden.

**Design:** Retrospective survey study.

**Participants and Setting:** Soccer players (*n* = 1,030) from 55 Swedish elite soccer teams. Questionnaires were completed prior to the start of the 2017 season.

**Assessment of Risk Factors:** Player history of soccer-related concussion (SoRC), symptoms and management following a SoRC were evaluated.

**Main Outcome Measures:** Before the start of the season the players completed a baseline questionnaire assessing previous concussions. The Sports Concussion Assessment Tool 3 was included with regard to symptom evaluation.

**Results:** Out of 993 responding players 334 (34.6%) reported a previous SoRC and 103 players (10.4%) reported a SoRC during the past year. After sustaining a SoRC, 114 players (34.2%) reported that they continued their ongoing activity without a period of rest, more commonly female (44.9%) than male players (27.7%; *P* = 0.002). Symptom resolution time was 1 week or less for 61.3% of the players that reported having persisting symptoms. A positive correlation was observed between number of previous concussions and prevalence of three persisting symptoms: fatigue (*P* < 0.001), concentration/memory issues (*P* = 0.002) and headache (*P* = 0.047).

**Conclusion:** About 35% of male and female elite soccer players in Sweden have experienced a previous SoRC, and about 10% experienced a SoRC during the last year. Female players continued to play after a SoRC, without a period of rest, more often than males. A higher risk of persisting symptoms was observed in players with a history of multiple concussions.

## Introduction

The most recent definition of a sports-related concussion states that it is a subset of mild traumatic brain injury (mTBI) that is induced by a blow either directly to the head or to another part of the body with the force being transmitted to the head (1). A concussion results in a short-lived disturbance of brain function that causes various symptoms and clinical signs typically lasting a few weeks but that in some cases can be more persistent and delay return to work, school and/or play (1).

Sports-related concussions account for about 5–9% of all sports injuries (2–4). A history of multiple concussions increases the risk of sustaining another concussion. Multiple concussions have been said to be associated with adverse health outcomes, slowed recovery of neurological function, and for a subset of individuals they may represent a risk of persistent post-concussion syndrome and plausibly chronic traumatic encephalopathy (5–8). Contact/collision sports, including soccer, have a higher incidence of concussions than non-contact sports, e.g., baseball and gymnastics (9). In addition, females have a higher risk of sustaining a concussion in sports in which the rules are the same for men and women (i.e., soccer, basketball, baseball/softball) (9–12).

Soccer is the world's most popular sport, with about 265 million players, 38 million of which are registered players^1^ Concussions represent 1–5% of all soccer-related injuries, with an incidence of 0.004–2.44 concussions per 1,000 player-hours (4, 9, 10). The concussion incidence for female soccer players is twice as high as for their male counterparts (11, 12). Possible reasons for this difference in concussion susceptibility, among others, may be a difference in neck strength, head acceleration when heading the ball and in willingness to report a concussion, to mention a few (13–16).

To our knowledge there are few studies regarding soccer-related concussions (SoRC), concussion symptoms and recovery in large populations of players of both males and females in a large number of elite teams. The main aim of the present study was to study the occurrence of previous concussions and concussion symptoms among soccer players and we hypothesized there could be important sex differences. More specifically, we also evaluated what kind of acute and persistent symptoms the players suffered from, the symptom resolution time as well as the management of the concussion.

## Methods

### Study Design and Participants

A descriptive study of concussion history, symptoms and management as well as of preseason, baseline symptoms evaluated among elite soccer players in Sweden.

In Sweden, there are about 350,000 soccer players over the age of 15^2^ There are 58 teams in the first and second soccer leagues for men and women in Sweden: 16 teams each in the first and second leagues for men, 12 and 14 teams in the first and second leagues for women, respectively. Teams were recruited at the annual conference for soccer medicine in Stockholm in January 2017 or through email/telephone. All 58 teams were asked to participate in the study. Players were only included if they were enrolled in a participating team from the start of the 2017 season; players joining the team later than that were not included in the study.

Individual response rate was calculated based on an estimated number of 22 players per team.

### Questionnaire Design

A baseline questionnaire was designed based on questions and evaluations from the 3rd edition of the Sports Concussion Assessment Tool (SCAT3) and was available for each team in both Swedish and English.

Demographic information was gathered on each player's age, years played at senior soccer level and medical background [migraine/headache diagnosis, ADHD (attention-deficit/hyperactivity disorder)/learning disabilities/dyslexia, depression/anxiety/other psychiatric diagnosis, medication use].

Information was also obtained regarding previous concussions, SoRCs as well as head injuries that required hospitalization for more than 24 h or where a brain hemorrhage had been visualized. A sports-related concussion was defined using the 4th consensus document for concussion in sport and presented in the questionnaire (17).

We specifically evaluated the history of SoRC regarding the acute symptoms (unconsciousness, amnesia, dizziness, nausea, and confusion) as well as the management (if the player had been removed from play or not at the time of the SoRC, if medical attention was sought, head imaging, and admission to the hospital undertaken). We also assessed persisting symptoms (headache, concentration and memory issues, dizziness and nausea) and consequences, (absence from full contact play) lasting more than 24 h.

The questionnaire included the SCAT3 with regard to symptom evaluation. SCAT3 covers 22 symptoms and uses a scale of 0–6 where 0 is classified as “none” and 5–6 as “severe.” The scoring generates two total scores: total number of symptoms (max score 22) and symptom severity score (max score 132).

### Data Collection and Exclusions

Questionnaires were sent electronically to members of the medical teams in February 2017. The 2017 competitive soccer season started in April 2017. The questionnaire was completed by the players prior to the start of the season and while the players were in a resting state. The authors extracted and anonymized all data from the completed questionnaires.

Questions not answered were excluded. When players did not to answer a question, it was recorded as a “missing answer” (Tables 1, 2). Missing answers on the questions regarding symptoms from SCAT3 were registered as 0 when calculating the total number of symptoms and the symptom severity score. Multiple answers on a question were registered as the highest score or longest time interval that had been marked. Percentages calculated for each question and group are based on total number of answers on that specific question (excluding missing answers).

**Table 1:** Participant demographics.

**Characteristics**	**Total** ***n* = 1030**	**Women** ***n* = 402**	**Men** ***n* = 628**	***P***	**Missing answers (*n*)**
Age, years^*^	24 (4.6)	22.5 (4.2)	24.9 (4.6)	<0.001	0
Years active on senior level*	7.4 (4.6)	7.2 (4.3)	7.5 (4.8)	0.670	58
Migraine/headache diagnosis^†^	208 (21.4)	98 (26.7)	110 (18.2)	0.003	60
Learning disabilities/ADHD^†^	31 (3.1)	13 (3.4)	18 (2.9)	0.709	40
Depression/anxiety^†^	27 (2.7)	16 (4.1)	11 (1.8)	0.029	14
**History of head injuries**^†^					
Head injury requiring hospitalization	22 (2.2)	10 (2.6)	12 (2.0)	0.659	34
Concussion	395 (39.8)	155 (40.1)	240 (39.7)	0.947	38
SoRc	344 (34.6)	130 (33.6)	214 (35.3)	0.585	37
SoRc during the last year	103 (10.4)	42 (10.9)	61 (10.1)	0.749	37

*Mean age, medical background and previous head injuries including soccer-related concussions among elite soccer players in Sweden. The denominator varies with missing answers*.

* *Reported as mean (SD)*.

*Reported as frequency and (%). Percentage was calculated based on total number of answers for each question, excluding missing answers*.

**Table 2:** Concussion symptoms and management.

**Acute concussion symptoms^†^**	**Total** ***n* = 335**	**Women** ***n* = 127**	**Men** ***n* = 208**	***P***	**Missing answers (*n*)**
Unconsciousness	126 (40.1)	31 (25.2)	95 (49.7)	<0.001	21
Amnesia	130 (40.4)	38 (30.4)	92 (46.7)	0.004	13
Dizziness	264 (82.0)	110 (88.7)	154 (77.8)	0.017	13
Nausea	190 (59.2)	85 (68.5)	105 (53.3)	0.007	14
Confusion	132 (42.9)	52 (44.1)	80 (42.1)	0.813	27
Concussion management^†^					
Continued activity at time of SoRC	114 (34.2)	57 (44.9)	57 (27.7)	0.002	2
Sought medical care	159 (47.9)	65 (51.6)	94 (45.6)	0.309	3
Head imaging	56 (17.9)	21 (17.2)	35 (18.4)	0.880	23
Admitted to hospital	41 (12.4)	12 (9.5)	29 (14.2)	0.233	5

*Acute concussion symptoms and management. Frequency of acute concussion symptoms and management of a SoRC among elite soccer players in Sweden. The denominator varies with missing answers*.

*Reported as frequency and (%). Percentage was calculated based on total number of answers for each question, excluding missing answers*.

*SoRC, soccer-related concussion*.

### Statistical Analysis

Statistical tests were performed with IBM SPSS Statistics (IBM Corp. Released 2017, IBM SPSS Statistics for Macintosh, Version 25.0, Armonk, NY, USA).

Means and standard deviations (SDs) were used for parametric data (age, years on senior level of soccer); median and interquartile range (q1 – q3) were used for non-parametric data (SCAT3 total number of symptoms and symptom severity score). The chi-square test or Fischer's exact test were used to compare categorical variables between groups. ANOVA or Mann-Whitney U-test were used to compare continuous variables between groups. Pearson correlation test was used to evaluate correlation between number of previous concussions and prevalence of persisting symptoms. The significance level was set at *P* < 0.05.

## Results

### Participating Players

Fifty-five of 58 eligible teams (94.8%) participated in the present study. A total of 1,030 players, 402 women (39.0%) and 628 men (61.0%), participated in the study. Estimated individual response rate was 80.7%. Demographic data and background information are presented in Table 1.

### Previous Concussions

A previous concussion of any cause was reported by 395 players (39.8%). A previous SoRC was reported by 344 players (34.6%). Of these 344 players, 189 players (54.9%) reported one previous SoRC, 150 players (43.6%) two to five previous SoRCs and five players (1.5%) six or more previous SoRCs. Ten percent of the players (103 players) reported a SoRC during the past year. No sex differences were found in the frequency of previous concussions or previous SoRCs (Table 1).

### Baseline SCAT3 Symptom Evaluation

**Figure 1:**
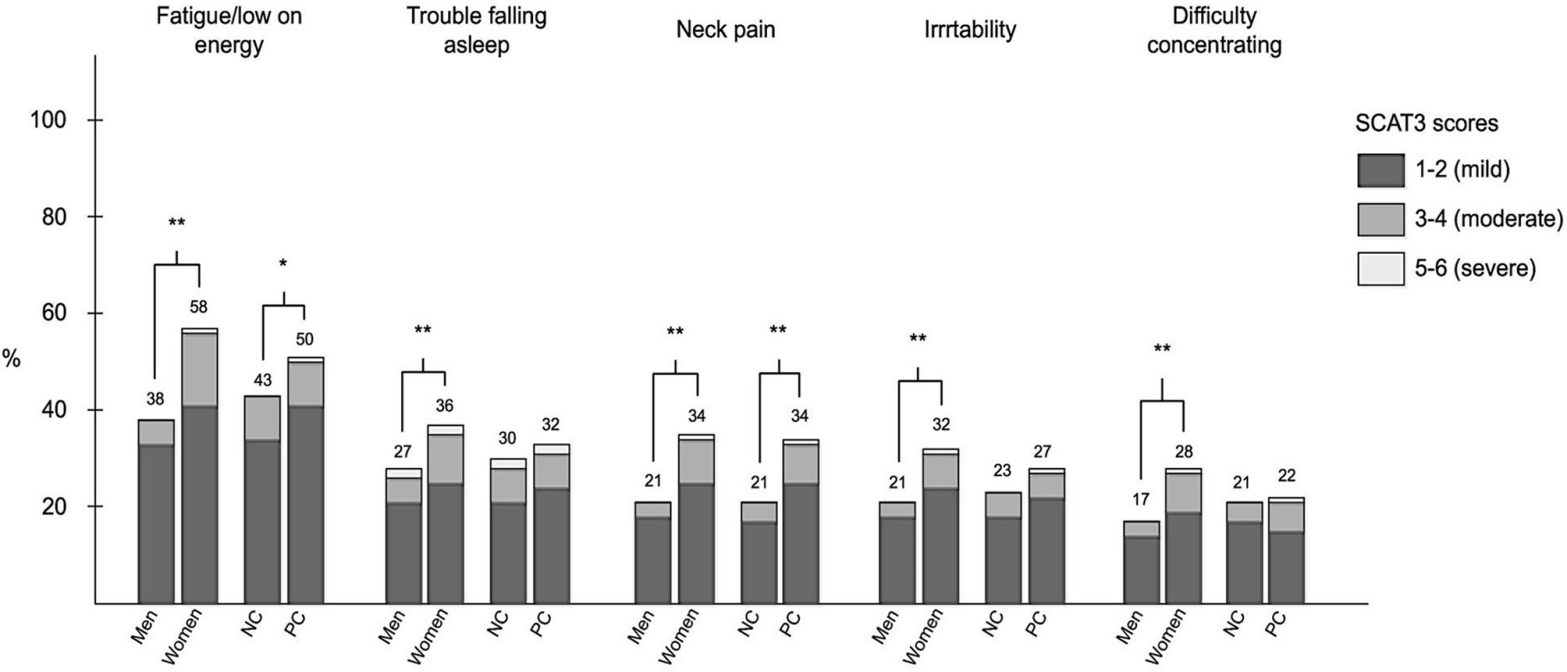
Frequency of the five most commonly reported symptoms in elite soccer players in Sweden evaluated with the Sports Concussion Assessment Tool-3 (SCAT3) and the frequency of symptom scores (graded 1–6) among men, women, never concussed (NC), and previously concussed (PC) players. Numbers above bars show percentage of group (men/women/NC/PC) with symptom. *P < 0.05, **P < 0.01.

Fatigue/low on energy was the most commonly reported preseason, baseline SCAT3 symptom and was reported by 469 players (45.9%). The overall median number of symptoms was 2 (0–5), and the median symptom severity score was 3 (range 0–8). The five most commonly reported SCAT3 symptoms are displayed in Figure 1.

### Male vs. Female

Twenty-one of the 22 baseline SCAT3 symptoms (all except “difficulty remembering”) were more commonly reported among female than male players (*P* < 0.05) (Figure 1). Female players had higher total number of symptoms [median 3 (1–7)] than the male players [median 1 (0–4)] (*P* < 0.001). The symptom severity score was also higher among female players [median 5 (1–12)], than among male players [median 2 (0–6)] (*P* < 0.001).

### Previously Concussed vs. Never Concussed

Eleven of the 22 baseline SCAT3 symptoms were more common in previously concussed players than in those never concussed (*P* < 0.05) (Figure 1). Previously concussed players had a higher total number of symptoms [median 2 (0–6)], than never concussed players [median 2 (0–4)] (*P* < 0.001). The symptom severity score was also higher in concussed players [median 4 (0–10)], than in never concussed players [median 2.5 (0–7)] (*P* < 0.001).

### Concussion Symptoms and Management

Questions on acute and persisting symptoms (persisting for > 24 h) following the most recent SoRC were answered by 335 players.

At time of concussion, both unconsciousness and amnesia were reported by 40.0% of the players, and both symptoms were more common among male than female players (*p* < 0.001). The most frequently reported acute SoRC symptoms were dizziness (82.0%) and nausea (59.2%); both symptoms were more common among female players (*p* = 0.017 for dizziness, *P* = 0.007 for nausea, Table 2).

At the time of a SoRC, 114 players (34.2%) continued their ongoing activity in the game or practice without a period of rest. This was more common among female players (*P* = 0.002). Hospital care was sought by 47.9% of the players and 12.4% were admitted to hospital (Table 2).

**Figure 2:**
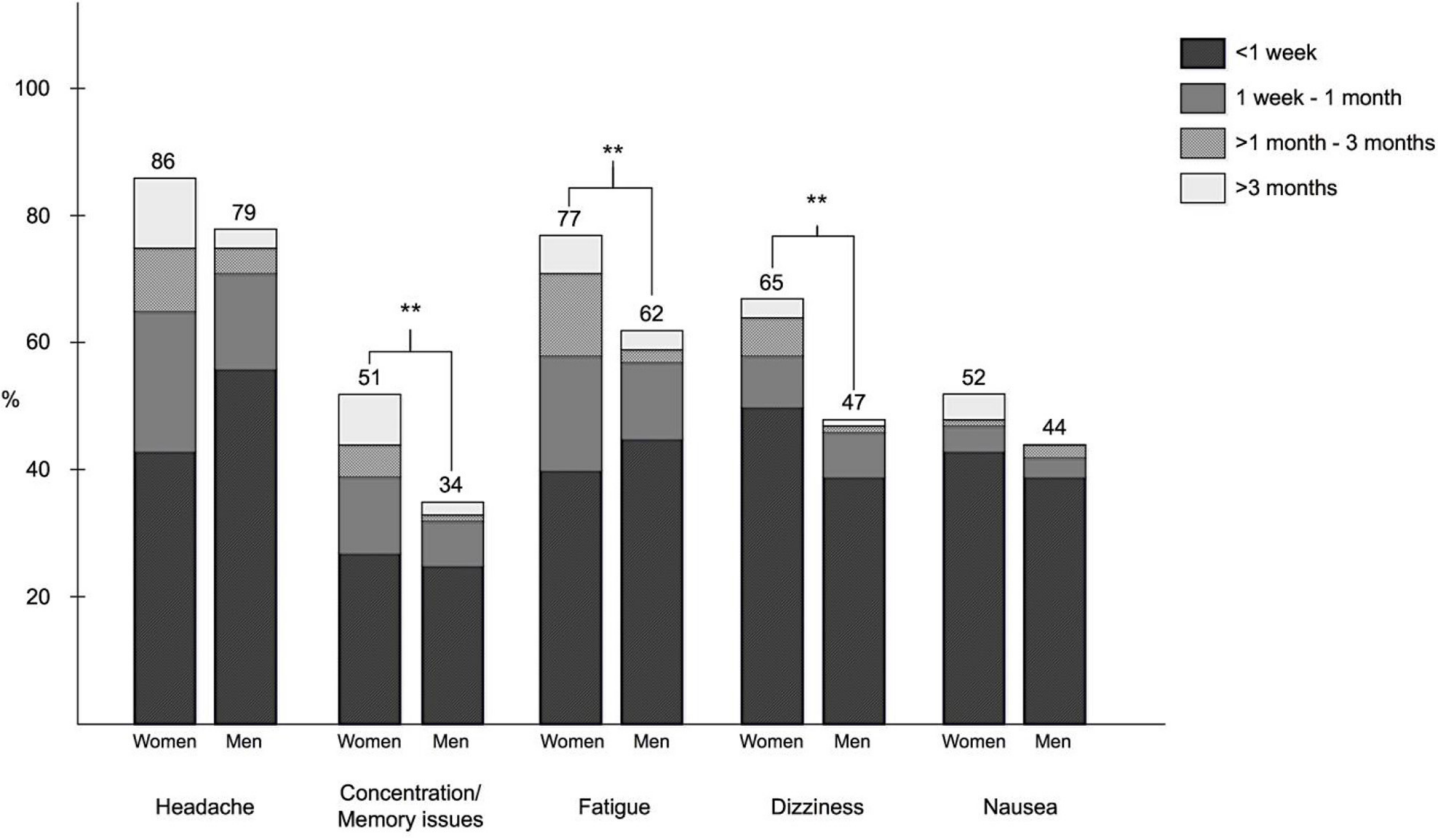
Frequency and duration of persisting symptoms reported after a soccer-related concussion (SoRC) among men and women. Numbers above bars show percentage of group (men/women) that reported the symptom. **P < 0.01.

At least one symptom (headache, concentration/memory issues, fatigue, dizziness and/or nausea) that persisted for > 24 h was reported by 295 players (88.3%). Of these symptoms, headache was the most common persisting symptom (reported by 271 players, 81.6%). Symptom resolution time was 1 week or less for 61.3% of the players. Symptoms persisted longer than 3 months in 8.5% of the players and longer than 1 year in 3.1% of the players. Concentration/memory issues, fatigue and dizziness were more frequently reported by female than male players (*P* = 0.003, *P* = 0.007, and *P* = 0.003, respectively, Figure 2).

**Figure 3:**
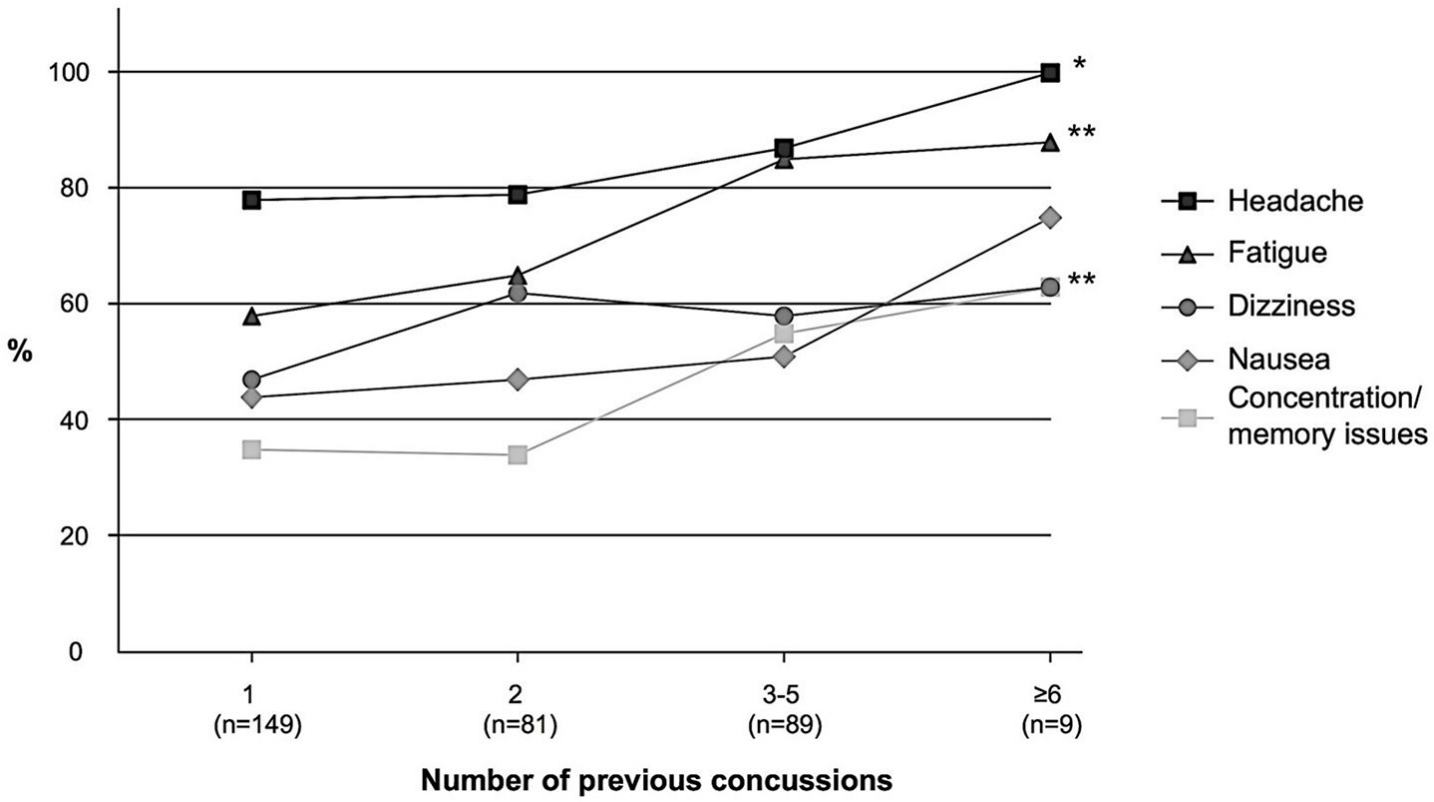
Prevalence of reported persisting symptoms (persisting > 24 h) following a soccer-related concussion in relation to number of previous concussions. *P < 0.05, **P < 0.01.

Absence from full-contact play, i.e., rest from full-contact game and/or practice play, was reported by 317 players (95.5% of the 335 players answering these questions). Of the players who had been absent from full-contact play, 283 players (89.3%) returned to full-contact play within 1 month after the concussion. A trend analysis demonstrated a positive correlation between number of previous concussions and prevalence of three of the persisting symptoms: fatigue (*P* < 0.001), concentration/memory issues (*P* = 0.002) and headache (*P* = 0.047, Figure 3).

## Discussion

The present study covered a large number of players (>1,000 players included) from almost all of the teams in the top four leagues in Sweden. About one-third of male and female elite soccer players in Sweden have experienced a previous SoRC, and about one in ten had experienced a SoRC during the last year. Female players continued to play after a SoRC, without a period of rest, more often than males. A higher risk of persisting symptoms was observed in players with a history of multiple concussions.

### Previous Concussions

The present study was a survey study based on the players' entire careers and an adequate measure of player hours/exposures for each player in the study population could not be estimated. The few previous studies with self-reported information have demonstrated that about 35–60% of the soccer players report a previous concussion (18–21). The present study demonstrated that about one-third of the players had sustained a previous concussion which is in accordance to previous reports.

In previous studies, women have been shown to be more prone to sustain sports-related concussions in soccer and other contact sports where the rules are the same for both sexes (9–12, 22–24). Possible contributing factors are differences in neck strength and head acceleration. Another contributing factor may be that concussions are not as often diagnosed in male athletes because they are less active in seeking medical care, which would lead to a lower concussion incidence (15). Contrary to previous research, our data do not suggest a sex difference in concussion history among elite soccer players in Sweden. These findings are contradictory. The study design (survey study) and the limitations associated with the study design (e.g., recall bias) could be a contributing factor. As mentioned above, it has previously been suggested that male players may be less prone to seek medical care. There is a possibility that male players may have reported previously undiagnosed concussions in our study. In addition, the mean age for male players was significantly higher than for female players in the present study. Assuming females and males start playing soccer at approximately the same age, the male group of players would have been exposed to high-risk soccer events for a longer period of time than the females. Furthermore, few studies have previously compared both females and males with the same method and with players at the same level, possibly contributing to contradictory findings. In a recent prospective study in a similar setting, no sex difference was observed when comparing concussion incidence among elite players (25).

### SCAT3 Symptom Evaluation

Similar to previous studies, fatigue/low on energy was the most commonly reported SCAT3 symptom (26, 27). SCAT3 symptoms were more common among females than males, which is in accordance with previous studies (28). The fact that half of the SCAT3 symptoms were also more common among previously concussed players compared to never concussed players indicates that concussion and symptom burden are associated. However, concussion symptoms are very non-specific and could have emerged for other reasons than the previous head injury. In the event of a concussion, it is therefore preferred that the medical staff evaluate how the symptoms develop over time. According to a recent study, a change from baseline of three or more of total number of symptoms and five or more of symptom severity score is considered “uncommon” (29).

### Concussion Management

According to consensus statements, an athlete with a suspected concussion should stop his or her activity immediately and rest for 24–48 h (1). Continuing to play could prolong symptoms and delay return to play (30). In contrast to current recommendations the present study reported that more than one-third of the players continued their ongoing activity immediately after the SoRC. The relatively high figures of non-adherence to guidelines for both men and women may have different causes. As acknowledged by recent consensus statements, symptoms may be vague and evolve over time and thus be missed at the time of impact. Another possible reason is players not reporting symptoms to the medical team to avoid being taken out of play, which would also result in the concussion being missed or diagnosed late (15). The fact that the medical staff has to act under a lot of pressure is another factor that could affect players not being taken out of play when they should. Following the men's World Cup in 2014, UEFA introduced new concussion procedures, which allows the referee to stop the game for up to 3 min, to allow the injured player to be assessed by the doctor. This was introduced in Sweden the following season.

Notably, there was a sex difference as almost half of the female players reported that they continued their activity after a SoRC. To our best knowledge, sex difference in same day return to play has not previously been reported. The reason for this observed difference can only be speculated upon. In the first and second Swedish soccer leagues for men, as well as the first soccer league for women, it is mandatory to have a medical team present during games. In the second soccer league for women however, there is no such requirement. The presence of a medical team during practices is not strictly regulated in any of the soccer leagues in Sweden. However, the presence of a medical team at practices is most likely higher in men's than women's soccer due to financial reasons. Compared to female leagues, men's teams have higher revenue, mainly due to higher earnings from for instance sponsoring, ticket-sales and broadcasting contracts. This is a likely explanation for the more common presence of the medical teams at practices in men's teams.

### Concussion Symptoms

Unconsciousness and amnesia were more common among males than females. Dizziness was the most commonly reported acute symptom and headache the most commonly reported persisting symptom. This is in accordance with previous studies reporting similar findings (9, 31–33).

Symptom resolution time was a week or less for the majority of players in the present study, which is similar to previous studies on concussion recovery (10, 24, 32, 34–36). Persistent symptoms could be detrimental to a player. In the present study almost 10% reported that their symptoms lasted longer than 3 months. We also found an association between the number of previous concussions and the incidence of persisting symptoms. The greater the number of concussions experienced in the past, the more likely the player was to experience fatigue, concentration/memory issues and headache after their most recent SoRC. Multiple concussions have previously been associated with slow recovery, memory impairment and poorer mental health (5–7, 37). This pinpoints the need for increased awareness of the risks with concussions in sport. The present study further emphasizes the negative effects of multiple concussions.

Even at professional level, athletic activity is healthy and leads to a reduction in all causes of death below the age of 70 (8). However, the same study also demonstrated that retired soccer players have higher mortality from neurodegenerative disease than matched controls, most probably due to repeated concussions and other head impacts during their career (8). Methods to protect players from sustaining concussions have been discussed more frequently in the last years. As a way to reduce incidence of sport-related concussions, the use of headgear was recently evaluated, although no reduction in concussion incidence was observed (24).

### Limitations

One main limitation of a survey study based on a questionnaire is the accuracy of self-reported concussions and the interpretation of the questions by the players. It is not unlikely that player recall lacks details about previous concussions, especially if they only had minor symptoms from the SoRC. Recall bias may lead to skewing of the data. In addition, opportunities and conditions to complete the questionnaire may have varied since it was each team's medical team that was responsible of administrating the questionnaire. Another limitation is inconsistent number of answers due to varying numbers of missing data.

When recruiting participants to survey studies, there is a possibility of selection bias. The fact that we managed to recruit almost all eligible teams in the top four leagues and had a relatively high estimated individual response rate probably reduced that risk. However, we were unable to access reliable data on the precise number of licensed players in each team at the start of the 2017 season. Therefore, the number of players who declined or missed the opportunity to participate could not be adequately calculated. Furthermore, players recruited to teams during the ongoing season were not included in the study.

## Conclusion

The present study aimed to evaluate concussion history and concussion-related symptoms among elite soccer players in Sweden. Similar to previous studies, the present study found that about one-third of the players reported having had a previous soccer-related concussion. Female players had a higher total number of baseline SCAT symptoms, as well as symptom severity scores. Contrary to previous studies, our data did not suggest a sex difference in concussion history (9–12, 22, 23). This contradictory finding could be due to different settings and methods in the present study. According to consensus statements a player should stop their activity immediately when sustaining a concussion (1). In contrast to these recommendations the present study revealed that more than one-third of the players in Sweden continued to play immediately after sustaining a concussion. This was more common among female players. Multiple concussions have previously been associated with slow recovery, memory impairment and poorer mental health (5–7, 37). Our data further emphasizes the negative effects of multiple concussions as the study demonstrated an association between number of previous concussions and the incidence of persisting symptoms. Thus, the present study has added important information on concussion history and concussion-related symptoms among soccer players.

## Data Availability Statement

The datasets generated for this study are available on request to the corresponding author.

## Ethics Statement

The Regional Research Ethics Committee at Uppsala University granted permission for the study (DNR 2017/151). Written informed consent was obtained from each player at the time of questionnaire completion.

## Author Contributions

SH, FV, NM, JJ, and YT designed the study and the questionnaire. SH extracted data from the questionnaires and performed the statistical analyzes. SH wrote the manuscript in consultation with FV, NM, JJ, and YT. All authors contributed to the article and approved the submitted version.

## Conflict of Interest

The authors declare that the research was conducted in the absence of any commercial or financial relationships that could be construed as a potential conflict of interest.

## Footnotes

^1^FIFA Big Count 2006: https://www.fifa.com/mm/document/fifafacts/bcoffsurv/bigcount.statspackage_7024.pdf.

^2^Swedish Football Association: https://fogis.se/om-svff/.
